# *Candida glabrata* Esophagitis: Are We Seeing the Emergence of a New Azole-Resistant Pathogen?

**DOI:** 10.1155/2014/371631

**Published:** 2014-12-03

**Authors:** Aze Wilson, Johan Delport, Terry Ponich

**Affiliations:** ^1^Division of Clinical Pharmacology and Division of Gastroenterology, Department of Medicine, London Health Sciences Centre, Western University, University Campus, 339 Windermere Road, Room C9-101, London, ON, Canada N6A 5W9; ^2^Department of Medical Microbiology, London Health Sciences Centre, Western University, Victoria Campus, 800 Commissioners Road E., Room B10-105, London, ON, Canada N6A 5W9; ^3^Division of Gastroenterology, Department of Medicine, London Health Sciences Centre, Western University, Victoria Campus, 800 Commissioners Road E., Room E1-317, London, ON, Canada N6A 5W9

## Abstract

*Background*. *Candida glabrata* (*C. glabrata*) has become a recognized pathogen in fungal esophagitis. A proportion of these isolates are azole-resistant which may have treatment implications. Variability in the prevalence of this organism exists in the limited data available. *Objective*. To determine the incidence of *C. glabrata* esophagitis in a North American hospital setting and to highlight factors that may predispose patients to this condition. *Methods*. Patient charts were collected from January 1, 2009 to July 30, 2011. Any charts of patients identified as having esophagitis with a positive fungal culture were reviewed for the species of *Candida* and the presence of factors that would predispose them to esophageal candidiasis. *Results*. The prevalence of *Candida* esophagitis based on culture was 2.2% (37 subjects). *C. glabrata* was the 2nd most prevalent pathogen identified (24.3% or 9 subjects). Of the *C. glabrata* cohort, all patients had at least one factor predisposing them to candidiasis. *Conclusion*. *C. glabrata* esophagitis makes up a large portion of the candidal esophagitis seen in hospital. *C. glabrata* infections were associated with at least one risk factor for candidal infection. Given its resistance to azole-based therapy, this may have treatment implications for how candidal esophagitis is approached by the clinician.

## 1. Introduction

Infections of the esophagus occur most commonly in immunocompromised patients such as those infected with human immunodeficiency virus and those receiving chemotherapy or immunosuppressive medications [1]. There are, however, esophageal infections that can occur in both the immunocompromised and the at-risk immunocompetent host (recent antibiotics, debilitated, aged, in-dwelling catheters).* Candida *species are one of the most commonly detected organisms in the setting of an esophageal infection [2]. Although* Candida albicans* (*C. albicans*) remains the most common cause of fungal esophagitis at many institutions, non-*albicans* species are increasingly associated with esophageal candidiasis [3]. Specifically, there have been an increasing number of reports identifying* Candida glabrata* (*C. glabrata*) as the causative agent in candidal mucosal and systemic infections [1, 4–10]. Despite this, publications on *C. glabrata* make up only a small proportion of publications on medically important fungal infections of the esophagus [11].

*C. glabrata *is yeast that belongs to the family Saccharomycetaceae and the genus* Candida*. Historically, it was thought to be primarily nonpathogenic; however, more recently, it has been shown to be highly opportunistic, achieving colonization through a series of adhesion proteins [11]. Unfortunately, very little is known about the pathogenesis and epidemiology of* C. glabrata*. What have emerged are reports of* C. glabrata*'s intermediate, dose-dependent susceptibility and 20% resistance rate against the azoles [12, 13]. With reports of resistance, an increasing prevalence of this organism could have an impact on the treatment of candidal esophagitis and how it is approached by the clinician.

The aim of this study was to determine the incidence of* Candida glabrata* esophagitis at a North American tertiary care centre and to highlight risk factors that may predispose patients to this condition.

## 2. Materials and Methods

This single centre, retrospective chart review was carried out in London, Ontario, Canada. Adult gastroenterology patient charts were included based on billing codes for esophageal biopsies and brushings taken from January 1, 2009, to July 30, 2011. All patients were at least 18 years of age. Outpatient and inpatient charts were included. Esophageal candidiasis was defined as the recovery of a* Candida* species from an esophageal biopsy or brushing.* Candida* esophagitis was confirmed endoscopically by the presence of typical sparse or coalescent white plaques. Neither the reason for performing the gastroscopy nor the patient's clinical symptoms were evaluated for the purposes of this study. The microbiologic results of each chart were reviewed. Any charts identified as having a positive fungal culture were further reviewed for the presence or absence of any risk factors that would predispose that patient to esophageal candidiasis as well as for the specific species of* Candida*. The study protocol was approved by the University of Western Ontario Health Sciences Research Ethics Board (protocol 18328E).

Prevalence data are presented as the arithmetic mean. Age and risk factor distribution were compared using a two-tailed *t*-test. A *P* value < 0.05 was deemed to be significant. All calculations were performed using Microsoft Corporation Analysis ToolPak, 2007.

Susceptibility patterns for the patients identified as having* C. glabrata* esophagitis in this cohort were unavailable. Susceptibility patterns for all* C. glabrata* cases at London Health Sciences from December 2011 to July 2012 were provided by the Division of Infectious Diseases at LHSC and reviewed.

## 3. Results

**Figure 1:**
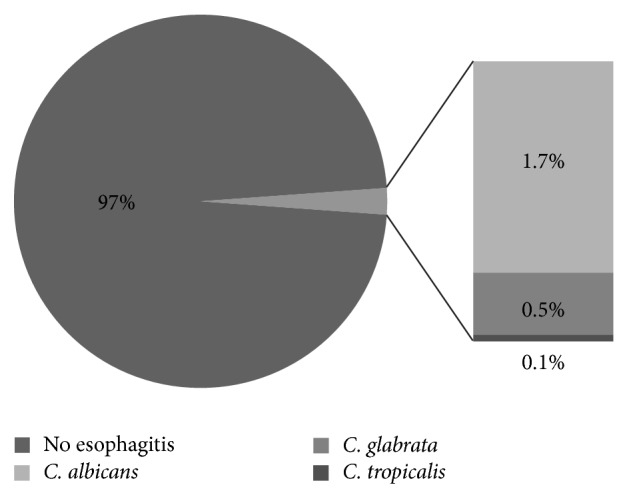
Prevalence of* Candida* esophagitis in an adult gastroenterology endoscopy cohort (2009–2011).* C.*:* Candida*.

**Figure 2:**
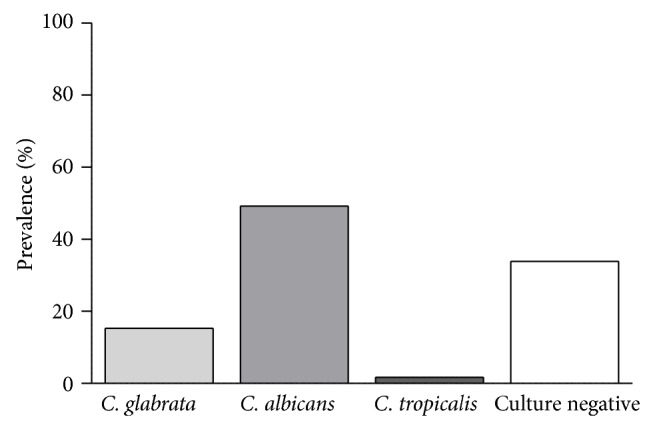
Distribution of* Candida* species in an adult gastroenterology cohort endoscopically and histologically identified as having* Candida* esophagitis.* C.*:* Candida*.

1701 charts were identified as meeting the inclusion criteria. Fifty-five subjects (3.2%) were recognized as having endoscopic findings consistent with* Candida* esophagitis (any species). However, the true prevalence of* Candida* esophagitis (any species) based on microbiologic culture was 2.2% (37 subjects) (Figure 1).* C. albicans* was implicated in the vast majority of infections (27 subjects or 73%) while* C. glabrata* was the next most prevalent (9 subjects or 24.3%), followed by* C. tropicalis* (1 subject or 2.7%) (Figure 2). Sixteen men (53%) and 14 women (47%) were affected. There were 6 cases where the subject had a mixed infection (5 mixed* C. glabrata/albicans* and 1 mixed* C. tropicalis/albicans*). With respect to the* glabrata*-infected subjects, ages ranged from 29 to 87, with a slightly younger mean age of 63 compared to the* albicans*-infected subjects, whose ages ranged from 23 to 99, with a mean age of 67 (*P* = 0.63). In the* C. glabrata* population, the majority of individuals were inpatients, while the* C. albicans *population were relatively equally distributed between in- and outpatients (Table 1).

The distribution of risk factors for candidal esophagitis did not vary significantly based on the species of* Candida *(*P* = 0.43). Of the* C. glabrata *cohort, all nine of the patients had at least one risk factor predisposing them to candidiasis compared with the* C. albicans* cohort, of which 62% of patients had at least one risk factor predisposing them to candidiasis. The mean number of risk factors per patient was 3, with a range of 1–8. Sixty-seven percent (6 subjects) of the* C. glabrata* cohort had a history of diabetes mellitus as well as pharmacological suppression of gastric acid production. The next most frequently cited factor predisposing to candidiasis was the recent use of antibiotic therapy (within the preceding 30 days) (44% or 4 subjects). Only one subject had been exposed to fluconazole in the preceding year.

Data regarding the azole-susceptibility pattern of* C. glabrata *was limited. Azole-susceptibility patterns of for the* C. glabrata* esophagitis cohort were unavailable. Data regarding the current azole-susceptibility patterns of all* C. glabrata* infections between December 2011 and July 2012 were limited. One hundred and eight* C. glabrata* cases were identified during this time period. Azole-susceptibility patterns for 20 of the cases were available. Of these cases, 3 were resistant, 1 was susceptible, and 16 were susceptible-dose-dependent (SDD); fluconazole MIC ≤ 32 mcg/mL.

## 4. Discussion

The prevalence of* C. glabrata* esophagitis at a North American tertiary care centre was 24% (9 subjects). The overall prevalence of* Candida* esophagitis of any species was 2.2% (37 subjects) based on microbiological culture. Both the latter and former frequencies are higher than what has been seen at other centres [1, 13–17]. Furthermore, the frequencies quoted at other centres tend to include all sites of candidal infection and are not limited solely to the esophagus. This suggests that the rates of glabrata infection of all body sites could be higher at our centre.

*C. glabrata*-associated esophagitis was associated with at least one risk factor predisposing to esophageal candidiasis in all patients. In particular, patients with diabetes and those admitted to hospital were more susceptible to this infection. Despite this, the mechanism for infection is largely unknown in this cohort of patients [18]. Furthermore, the risk factors predisposing an individual to* C. glabrata* esophagitis specifically are not well defined in the literature [18]. The presumption that these factors are similar to what is seen with* C. albicans* esophagitis continues to exist in the literature. Based on this study, this assumption holds true; however, given the retrospective nature of this review, some factors may not have been identified. It should also be noted that, despite the fact that candidal esophagitis is often seen in human immunodeficiency virus (HIV) infected individuals [19, 20], all of the patients included in this study were HIV negative. The clinical significance of this is unknown. Furthermore, as seen with previous antibiotic exposure and the selection for drug resistant bacteria, one could hypothesize that azole-resistant candidal species would be selected for with previous azole exposure. This was not seen in this population. Only one subject of the* C. glabrata* cohort had previous exposure to fluconazole, while none of the* C. albicans* had any azole exposure in the preceding year. From this study, azole-exposure cannot be cited as a risk factor for* C. glabrata* esophagitis. This may be a reflection of the low number of* C. glabrata* cases identified and may not be the case if a larger cohort of cases were analyzed.

A limitation of this study is that the antifungal susceptibilities of the identified* C. glabrata* esophagitis cases are not known. As previously mentioned, a reduced susceptibility, fluconazole MIC ≤ 32 mcg/mL (SDD), and a 20% resistance rate, fluconazole MIC ≥ 64 mcg/mL, have been seen with* C. glabrata* [12, 13]. Only limited data regarding the azole-susceptibility patterns of* C. glabrata* was known. The majority of the* C. glabrata* infections were azole-resistant or SSD. Given the relatively high prevalence at this centre compared with other centres, it would be beneficial to track the rates of azole-resistance more accurately. The Infectious Diseases Society of America recommends that esophageal candidiasis be treated empirically with fluconazole 200–400 mg daily for 14–21 days [12]. This may not be adequate for* C. glabrata* infections, where they recommend treatment with an echinocandin, unless azole-susceptibility has been demonstrated [12]. Empiric treatment of* Candida* esophagitis based on endoscopic findings is not an uncommon practice at this centre and may be seen at other centres as well [21]. This could lead to inadequate treatment of infection and could be selected for higher degrees of resistance.

## 5. Conclusion

*C. glabrata*-associated esophagitis makes up a large portion of the candidal esophageal infections seen in a North American hospital setting, higher than what has been previously quoted. A high degree of suspicion should be maintained for this infection in individuals presenting with risk factors similar to those that predispose to* C. albicans* infection, especially amongst patients admitted to hospital. Further investigation is needed to better characterize all the predisposing risk factors.

Furthermore, empirical treatment of esophageal* Candida* infections with fluconazole based on endoscopic appearance alone may not be effective for* C. glabrata*. This study highlights the need to take esophageal brushings and await culture results rather than proceeding with empiric treatment. It also highlights the need for clinicians to be aware of the* C. glabrata* azole-susceptibility patterns at their centre.

## Figures and Tables

**Table 1:** Factors associated with *Candida* species esophagitis.

Factor	*C. glabrata* (*n* = 9)	*C. albicans* (*n* = 27)	*C. tropicalis* (*n* = 1)
Demographics			
Age (range, mean)	29–87, 63.3	23–99, 67.1	84
Males	4 (44%)	13 (50%)	1 (100%)
Females	5 (56%)	14 (54%)	0 (0%)
Inpatient	7 (78%)	14 (50%)	1 (100%)
Outpatient	2 (22%)	11 (42%)	0 (0%)
Nursing home resident	1 (11%)	2 (8%)	0 (0%)
Comorbid conditions			
HIV	0 (0%)	0 (0%)	0 (0%)
Active cancer	2 (22%)	8 (31%)	0 (0%)
Diabetes	6 (67%)	9 (35%)	0 (0%)
Hypothyroidism	2 (22%)	2 (8%)	0 (0%)
Chronic kidney disease (on hemodialysis)	1 (11%)	1 (4%)	0 (0%)
Cirrhosis	1 (11%)	2 (8%)	0 (0%)
Motility disorder of the esophagus	0 (0%)	3 (12%)	0 (0%)
Splenectomy	1 (11%)	0 (0%)	0 (0%)
Medical therapy			
PPI therapy	6 (67%)	16 (58%)	0 (0%)
Immunosuppressive therapy	3 (33%)	7 (27%)	0 (0%)
Antibiotics (last 30 days)	4 (44%)	9 (35%)	1 (100%)
Indwelling catheter	2 (22%)	1 (4%)	0 (0%)
Azole exposure (last year)	1 (11%)	0 (0%)	0 (0%)
Social factors			
Current smoker	3 (33%)	7 (27%)	0 (0%)
EtOH use (>7/week)	2 (22%)	5 (19%)	0 (0%)

HIV: human immunodeficiency virus; EtOH: alcohol; PPI: proton-pump inhibitor.
